# Human *in vivo* talocrural contributions to ankle joint complex kinematics during walking, running, and hopping

**DOI:** 10.1016/j.heliyon.2024.e41301

**Published:** 2024-12-19

**Authors:** Anja-Verena Behling, Lauren Welte, Michael J. Rainbow, Luke Kelly

**Affiliations:** a School of Human Movement and Nutrition Science, The University of Queensland, Brisbane, Australia; b Department of Mechanical and Materials Engineering, Queen's University, Kingston, Canada; c Department of Mechanical Engineering, University of Alberta, Edmonton, Canada; d Department of Biomedical Engineering, University of Alberta, Edmonton, Canada; e Australian Centre for Precision Health & Technology, Griffith University, Gold Coast, Australia

## Abstract

The human ankle joint complex, consisting of calcaneus, talus, and tibia, is often simplified as a single functional ankle joint, neglecting the motion of the talus. Understanding the individual contributions of the talus and calcaneus is crucial for comprehending ankle joint complex function in healthy populations, and alterations in function that may exist in clinical conditions.

To achieve accurate bone kinematics, high-resolution biplanar videoradiography was used with participants engaged in walking and running (n = 9) and hopping (n = 9) with no overlap in participants. The rotation axes for the calcaneus and talus were analysed relative to the tibia over the ankle joint dorsi-/plantar flexion phases. Contributions of the talocrural joint to overall ankle joint complex function were measured by comparing the range of motion (talus relative to calcaneus).

Most of the sagittal plane motion in the ankle joint complex (80%) occurred in the talocrural joint, with the subtalar joint contributing 20%. Rotation of the calcaneus about the tibia dominated frontal and transverse plane motion during hopping. Although surprisingly large ranges of talus motion were also observed in these planes (>5deg), indicating notable inter-subject variability and that the talocrural joint is not a simple hinge joint.

The study highlights the importance of directly quantifying talus motion to understand ankle joint complex function. The results showed opposing talus and calcaneus movements for many participants during different locomotor tasks, which may influence the magnitude and distribution of subtalar joint loading. In addition, the large out-of-sagittal plane movements and inter-subject variability in talus and calcaneus motion may further emphasize the need for personalised models to investigate treatments for ankle pathologies.

## 1 Introduction

The human ankle joint complex and its associated articulation, play an essential role in our capacity to walk and run in an upright manner [1]. The talocrural joint is typically conceptualized as a hinge joint that mainly moves in the sagittal plane (flexion/extension) [2]. In contrast, the subtalar joint is usually considered to primarily rotate in the frontal and transverse planes (inversion/eversion and abduction/adduction) [3]. If mathematically combined, the motion of the talocrural and subtalar joints result in overall ankle joint complex motion (calcaneus relative to tibia). Despite the importance of this joint complex, we lack a clear understanding of how the talocrural, and subtalar joints contribute to overall ankle joint complex function.

Traditionally, the ankle joint complex has been oversimplified by considering the entire complex as one joint, rather than dividing it into the talocrural and subtalar joints. Our oversimplified understanding of the ankle joint complex is likely due to technological and methodological limitations. The majority of approaches to assess ankle joint complex function in research and clinical contexts involve using skin-mounted markers and optical motion capture systems to track the markers and subsequently estimate bone motion, based on the movement of markers attached to the skin overlaying the bone of interest [[4], [5], [6]]. Unfortunately, this approach cannot accurately track the motion of the talus, due to the deep location of this bone (located between the tibia and fibula) preventing markers from being applied to the skin overlaying its surface. Therefore, previous motion capture procedures usually rely on the motion of the calcaneus relative to the tibia as metric for ankle joint complex motion.

Intracortical bone pin studies overcome the limitations of optical motion capture, by directly tracking the motion of markers attached to pins that are surgically inserted into individual bones of interest. In a series of landmark papers [[7], [8], [9], [10]], this invasive approach has provided highly novel insights (large inter-subject variability in talocrural and subtalar joints). However, the approach is limited by the invasive nature of the technique, and that bone coordinate systems were aligned to a global reference frame (normalized) and were not based on anatomical or morphological considerations [11].

Dynamic musculoskeletal imaging approaches, such as videoradiography allow non-invasive measurement of skeletal motion [1,[12], [13], [14], [15], [16], [17], [18], [19]]. Foot bone joint motion was reported relative to various reference bones (typically, the proximal bone was selected as the reference bone) often using traditional Euler-angle based approaches [12,[14], [15], [16], [17],[19], [20], [21], [22]]. While commonly applied in human biomechanics research, this approach may limit understanding of the interactions between adjacent joints of a multi-articular complex (e.g., the ankle joint complex). First, the Euler-axis approach does not represent orthogonal rotations and treats each joint (bone-pair) as independent of all adjacent joints with common bones. It also requires a specified rotation sequence for each bone-pair, which might vary across bones within the same joint complex.

Helical axis of motion calculations have previously been combined with a contribution approach to understand complex osseous interactions within the human wrist [[23], [24], [25], [26]]. Given the similarities in structure and function complexity between and the wrist and the ankle, a combined contribution approach may deliver unique insights into the interactions between individual bones and joints to overall ankle joint complex function. The contribution approach requires a common reference frame and offers information on inter-joint complex coordination that would otherwise remain unknown using traditional Euler-angle based approaches (e.g., normalization to a global position or specify a rotation sequence). For example, even if the overall motion remains consistent across conditions (e.g., pre- and post-treatment), the distribution of motion among the joints may vary, highlighting the importance of a nuanced understanding of the complex function and individual joint variability within the ankle joint complex.

This study aims to determine the relative contribution of the talocrural joint (talus relative to tibia) to overall ankle joint complex (calcaneus relative to tibia) motion *in vivo.* Therefore, we collected biplanar videoradiography (BVR) data during walking, running, and hopping to quantify the helical axes of motion of the calcaneus and talus, relative to a common reference bone (tibia), resolved in a morphological coordinate system of the tibia. The three locomotion tasks reflect our most common modes of locomotion (walking and running) and a task that maximizes dynamic ankle range of motion (hopping). We adapted the contribution approach for the ankle joint complex (based on wrist literature [[23], [24], [25], [26]]) to determine the relative contribution of an individual joint (talus relative to tibia) to the global joint complex (calcaneus relative to tibia) motion *in vivo*. We hypothesized that the contributions to motion of the ankle joint complex (calcaneus relative to tibia) will be divided such that the talocrural joint will contribute predominantly to sagittal plane motion and the subtalar joint will contribute predominantly to transverse and frontal plane motion. In addition, we have divided each ground contact phase according to the dominant sagittal plane of motion patterns (dorsi- and plantar flexion phase) according to previous findings of these two phases being energetically and kinematically different [[27], [28], [29]]. We further hypothesized differences between the dorsi- and plantar flexion phase regarding each locomotion task's contributions and range of motions.

## 2 Methodology

### 2.1 Overview and participants

Kinematic data were combined from two separate data collections and study protocols for the purpose of this analysis, with no participants were overlapping between studies. Ethical approval was provided by the Institutional Review Ethics Board of Queen's University (MECH-060-17 and MECH-063-18) and written informed consent was obtained from each participant prior to data collection.

Overground walking and running trials at self-selected speeds (mean ± std: walking speed 1.4 ± 0.3 m/s; running speed 3.0 ± 0.5 m/s) were obtained from nine healthy participants (5 females; mean ± std; mass 76.1 ± 15.8 kg; height 171.1 ± 15.8 cm; 20.0 ± 6.8 years) with no recent history of lower limb injury. The participants’ right legs were imaged using biplanar video radiography (125 Hz walking, 250 Hz running, range of 70–80 kV, 100–125 mA) and wearing minimalist shoes (Prio, Xero Shoes, USA). Participants were fitted with minimalist shoes to mitigate potential discomfort of barefoot running and any potential adjustments to their natural gait patterns. Given the minimal cushioning of the shoes, we anticipated that they had negligible influence on bone motion. Force plate data were collected simultaneously (1125 Hz, AMTI Optima, AMTI, USA) and was automatically synchronized to the BVR via a trigger step function. The stance phase of a single right foot per condition was analysed from the three collected trials. The field of view for the BVR system is relatively small, so the foot bones of interest are not always visible for the entire ground contact period. Therefore, each trial was screened for maximal completeness (Supplement Fig. S1).

Unilateral hopping data was obtained from nine healthy subjects (5 females; mean ± std, mass 71.3 ± 14.5 kg; height 171.4 ± 10.7 cm; 27.6 ± 7.4 years) with no history of lower limb injury. Participants hopped on their right leg while matching their hopping frequency to a metronome of 156 bpm, which controlled for similar hopping cycle durations and therefore, indirectly also for hopping heights across trials and participants (hopping height approximated via vertical posterior pelvis marker displacement: 9.9 ± 1 cm). Barefoot hopping trials were collected using BVR (125 Hz, range of 70–80 kV, 100–125 mA), and force plate data were collected simultaneously (1125Hz, AMTI Optima, AMTI, USA) and manually synchronized to the BVR using optical motion capture (Qualysis Track Manager, Gothenburg, Sweden). Within each trial, three hops with the entire foot in the field of view were processed. Hopping provided an excellent opportunity to capture the entire foot bones throughout multiple cycles without leaving the small field of view of the BVR system.

### 2.2 Data processing: kinematics derived from biplanar videoradiography

A computed tomography scan (CT, protocol settings were the same between both studies 120 kV, 60 mA, model: Lightspeed 16, Revolution HD, General Electric Medical Systems, USA) was obtained from each participant's right foot while they lay in a prone position (average resolution: 0.336 x 0.336 × 0.625 mm; average scanned volume 172.0 x 172.0 × 309.0 mm with an average tibia length of 11.7 ± 0.9 cm). Tibia, calcaneus, and talus bones were segmented (Mimics 24.0, Materialise, Belgium). Bone surface meshes and digitally reconstructed radiographs (DRRs) were generated from the CT scans [30].

BVR is a musculoskeletal imaging modality allowing for highly accurate and precise foot bone tracking across activities [15]. The processing pipeline for foot BVR data can be found elsewhere [31]. Briefly, the high-speed cameras were calibrated using a custom calibration cube, and the images were undistorted [32] using X-ray-specific software (XMALab, Brown University, USA) [33]. The translation and orientation of the tibia, calcaneus, and talus were measured by matching the DRRs to biplane radiographs using custom software (Autoscoper, Brown University, USA) [30]. Tracking accuracy was ensured with a rigorous user-training protocol, based on a publicly available training set [34] until the bones can be matched within a 1 mm translation and a 2° rotation error compared to the gold standard of processing biplanar videoradiography data (tracking tantalum beads embedded in the bone of interest) [32,35]. Custom MATLAB scripts were used to perform all further calculations and analyses, including statistical analyses (R2023a, MathWorks Inc., Natick, USA).

We implemented a previously established shape-based coordinate system for the tibia [36] with its primary axes determined by a cylinder fit through the distal tibia dome. The coordinate systems for the calcaneus and talus were inertia based, but as we use a helical axis approach, the choice of coordinate systems for the moving bones of interest is irrelevant. The axes were re-labelled such that the *x*-, *y*- and *z*-axes more closely represented dorsiflexion, inversion, and adduction, respectively, with reference to the right tibia and right-hand rule [36,37].

Due to locomotion specific variations in kinematic and kinetic behaviour of the ankle joint complex, we have adopted locomotion mode specific events to define our analyses. Common to all locomotion modes, we decided to focus on two different phases (ankle joint complex dorsi- and plantar flexion) due to the energetically and kinematically distinct patterns [[27], [28], [29]]. All modes are defined by an initial touchdown event, a transition event, and a take-off event, but the definition of when these events occur, varies among locomotion modes. For hopping, foot touchdown and take-off events were defined using a 15 N threshold in the vertical ground reaction force (GRF). The transition point was determined as the active peak of the vertical GRF. This approach is not ideal for walking and running, as some participants displayed a "foot slap" (ankle plantar flexion) during early ground contact, which might distort the magnitude of rotation about (and orientation of) the helical axis. Additionally, there was no singular active peak in the vertical GRF. Hence, we took a kinematic approach for walking and a mixed approach (kinematic and kinetic) for running to define our gait events. For both walking and running, we calculated Euler angles for the ankle joint complex (calcaneus relative to the tibia, XYZ-sequence) for the support phase (determined with a 15N threshold in vertical GRF). For walking, the touchdown event was defined as the maximal ankle plantar flexion in the first half of the support phase (i.e., initiation of ankle joint complex dorsiflexion). The transition point was defined as the maximal ankle joint complex dorsiflexion angle, and take-off was the same as for hopping (15N threshold in vertical GRF). For running, the touchdown was determined in the same way as for walking (initiation of ankle joint dorsiflexion), the transition point was at the zero-crossing of the anterior-posterior GRF and toe-off was determined the same as for walking and hopping. The dorsiflexion phase describes the phase from touchdown to the transition point and the plantar flexion phase from the transition point to toe-off for each locomotion task.

**Fig. 1:**
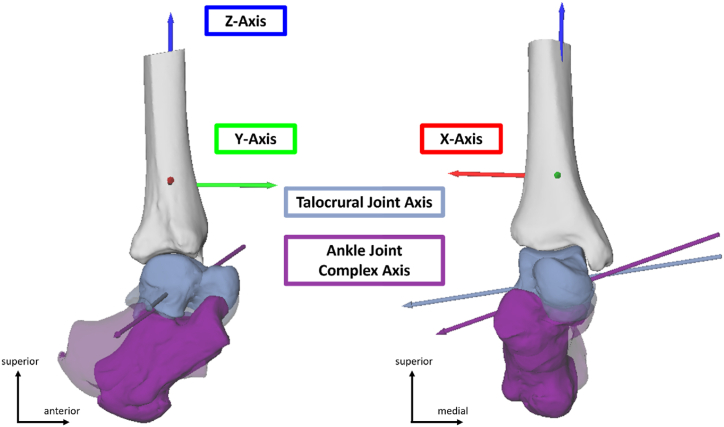
Right leg; lateral view (left) and frontal view (right). Example helical axes of motion for the dorsiflexion phase during hopping (n_Participant_ = 1; n_Trials_hopping_ = 1) for the talocrural joint (blue grey) and the ankle joint complex axis (purple) relative to the tibia. Helical axes are indicated as arrows and follows the right hand rule. The tibia coordinate system is indicated in red (x-axis), green (y-axis) and blue (z-axis). Ankle joint complex bones (talus and calcaneus) are shown in two postures: shaded bones indicate the touchdown position (plantarflexed) while the solid bones indicate the transition point position (dorsiflexed).

Finite helical axes were calculated for the talocrural joint (talus relative to the tibia) and calcaneus relative to the tibia (ankle joint complex) and resolved in the tibia coordinate system [38] (Fig. 1). Hence, each helical axis represents the change in position and orientation of the moving bone between two time points relative to its reference bone. Each helical axis can be described by its translation along and rotation about the axis. Each gait phase corresponds to one helical axis, which was calculated from touchdown to the transition point and the transition point to toe-off, respectively.

### 2.3 Analysis

The total rotation and the motion per plane (product of total rotation and the unit vector per direction) were evaluated in this study. If the total rotation for a joint was less than 5°, the helical axis was considered unstable and no kinematics per plane were calculated [39]. The kinematics of the ankle joint complex (talocrural and ankle joint complex) in the dorsi- and plantar flexion phase across all trials and subjects were reported as the average ± 1 standard deviation.

To investigate how much the talocrural joint (and indirectly the subtalar joint) contributes to ankle joint complex motion, percentage contributions of the talocrural joint motion were reported relative to the sagittal plane motion and total rotation of the ankle joint complex.

To test whether there were significant differences in talocrural joint and ankle joint complex kinematics, paired t-tests compared the total rotation magnitudes and motion in each plane between the talocrural joint and ankle joint complex.

To test whether dorsi- and plantar flexion phase kinematics are significantly different, paired t-tests compared the total rotation magnitudes between phases for each joint respectively.

The significance level was set to 0.05 and corrected for multiple comparisons when required using a Bonferroni correction. The non-parametric equivalent of paired t-test was used whenever the data were not normally distributed. Importantly, the following statistical analyses and derived interpretations should be viewed as exploratory due to the small sample size. Within-subject designs were employed to focus on individual changes rather than group means.

## 3 Results

**Fig. 2:**
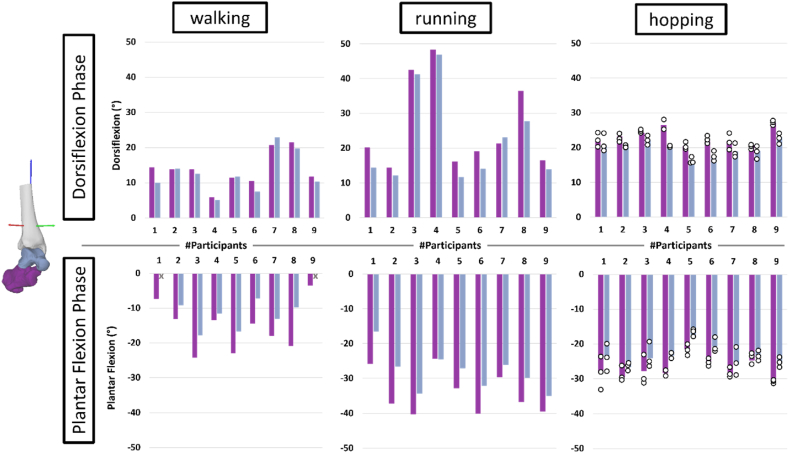
Sagittal plane kinematics for talocrural joint (blue grey) and ankle joint complex (purple) joint for all participants (n_Participants_ = 9; n_Trials_walking/running_ = 1; n_Trials_hopping_ = 3) in degrees. Positive and negative rotations are determined based on the right-hand rule (i.e., dorsiflexion is positive). The dorsiflexion phase is at the top row while the plantar flexion phase is in the bottom row. X indicates a missing value due to total rotation values of <5°. Subtalar joint motion can be inferred by the difference between the ankle joint complex and the talocrural joint. The white circles indicate individual trials for hopping.

As expected, participants also consistently showed ankle joint complex dorsiflexion during the dorsiflexion phase and plantar flexion during the plantar flexion phase, in every locomotion task (Fig. 2).

**Fig. 3:**
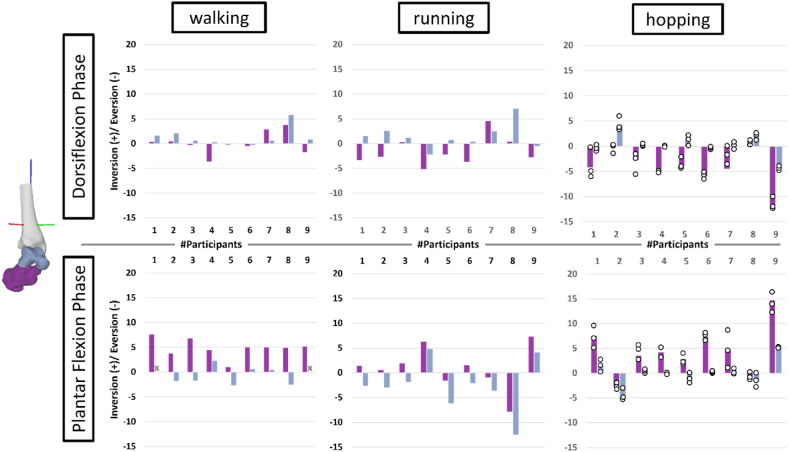
Frontal plane kinematics for talocrural joint (blue grey) and ankle joint complex (purple) for all participants (n_Participants_ = 9; n_Trials_walking/running_ = 1; n_Trials_hopping_ = 3) in degrees. Positive and negative rotations are determined based on the right-hand rule (i.e. inversion is positive). The dorsiflexion phase is at the top row while the plantar flexion phase is in the bottom row. X indicates a missing value due to total rotation values of <5°. Subtalar joint motion can be inferred by the difference between the ankle joint complex and the talocrural joint. The white circles indicate individual trials for hopping.

The magnitude and direction of rotations in the frontal and transverse planes displayed substantial variability within (only for hopping) and between individuals for each different locomotion task (Fig. 3, Supplement Figs. S2 and S3). Highlighting inter-participant variability, participants showed different gait patterns across both joints. For example, participant #4 displays synchronous talocrural and ankle joint complex eversion in the dorsiflexion phase of running, while participant #7 displays inversion for both joints in the dorsiflexion phase. And participant #2 displays talocrural joint inversion while the ankle joint complex is everting (and vice versa during the plantar flexion phase) during the dorsiflexion phase of running.

Between 65 and 95 % of the total rotation and sagittal plane motion occurred about the talocrural joint during walking, running, and hopping regardless the gait phase (Table 1).

**Table 1:** Percent contributions of the talocrural joint relative to the ankle joint complex motion in the sagittal plane and relative to the total rotation within each locomotion task represented as mean ± 1 standard deviation [minimum, maximum] for all participants(n_Participants_ = 9). Please note that a contribution >100 % is interpreted as the magnitude of the talocrural joint rotation being greater than the magnitude of ankle joint complex rotation. This suggests that the subtalar joint must be moving in the opposite direction to the talocrural joint.

TALOCRURAL CONTRIBUTIONS [%]		Walking	Running	Hopping
Dorsiflexion Phase	Total Rotation	93.2 ± 11.5 [76.3, 110.0]	83.4 ± 13.7 [68.3, 108.7]	82.3 ± 7.4 [73.3, 91.9]

Dorsi/Plantar Flexion	95.1 ± 17.0 [71.4, 110.5]	86.4 ± 12.4 [63.9100.6]	85.6 ± 6.0 [77.8, 92.7]
Plantar Flexion Phase	Total Rotation	65.5 ± 15.3 [42.9, 83.5]	81.4 ± 9.2 [63.0, 94.5]	82.2 ± 7.0 [82.4, 93.4]
	Dorsi/Plantar Flexion	67.4 ± 12.9 [46.8, 86.1]	82.3 ± 9.9 [71.4, 108.4]	85.8 ± 4.7 [80.2, 93.7]

The magnitude of total rotations, as well as sagittal and frontal plane motion were significantly larger in the ankle joint complex compared to the talocrural joint (p = 0.004; indicated with an asterisk in Table 2; individual total rotation magnitudes can be seen in Fig. S2). During running, the ankle joint complex total rotation and motion in the transverse plane were significantly larger than the talocrural joint rotations and motion. No statistical differences for range of motion metrics were found in walking.

**Table 2:** Range of motion of the ankle joint complex and talocrural joint (°) across locomotion tasks represented as mean ± 1 standard deviation (n_Participants_ = 9). Statistical significance between the ankle joint complex and talocrural joint is indicated with an asterisk (= 0.007) and a cross indicates statistical significance between the dorsiflexion and plantar flexion phases (α = 0.025) for each locomotion task. All significant comparisons had a p-value of 0.004.

RANGE OF MOTION [°]			Walking	Running	Hopping
Ankle Joint Complex	Dorsiflexion Phase	Total Rotation	14.3 ± 4.6	26.8 ± 12.6	24.6 ± 3.2∗†
		Dorsi (+)/Plantar Flexion (−)	13.7 ± 4.9	26.0 ± 12.8	23.1 ± 2.4∗
		Inversion (+)/Eversion (−)	0.1 ± 2.2	−1.6 ± 2.9	−4.3 ± 3.7∗
		Internal (+)/External Rotation (−)	2.9 ± 3.4	−4.4 ± 2.6	−4.8 ± 4.8
	Plantar Flexion Phase	Total Rotation	17.3 ± 6.0	35.4 ± 5.9∗	29.0 ± 3.8∗†
		Dorsi (+)/Plantar Flexion (−)	−15.2 ± 6.5	−34.0 ± 5.8	−27.2 ± 2.6∗
		Inversion (+)/Eversion (−)	4.8 ± 1.8	0.9 ± 4.1	4.6 ± 4.7∗
		Internal (+)/External Rotation (−)	4.3 ± 3.8	8.3 ± 3.2∗	5.8 ± 5.3
Talocrural Joint	Dorsiflexion Phase	Total Rotation	13.6 ± 5.3	23.1 ± 13.2	20.1 ± 2.2∗†
		Dorsi (+)/Plantar Flexion (−)	12.6 ± 5.6	22.7 ± 13.3	19.7 ± 2.2∗
		Inversion (+)/Eversion (−)	1.2 ± 1.8	1.4 ± 2.5	0.2 ± 2.3∗
		Internal (+)/External Rotation (−)	1.7 ± 2.9	−0.8 ± 2.6	−1.0 ± 2.9
	Plantar Flexion Phase	Total Rotation	12.6 ± 3.5	28.9 ± 5.8∗	23.7 ± 2.9∗†
		Dorsi (+)/Plantar Flexion (−)	−12.1 ± 3.6	−27.9 ± 5.3	−23.3 ± 2.7∗
		Inversion (+)/Eversion (−)	−0.8 ± 1.7	−2.5 ± 4.8	−0.13 ± 2.6∗
		Internal (+)/External Rotation (−)	−0.3 ± 2.7	3.8 ± 3.4∗	0.9 ± 3.2

During hopping, ankle joint complex and talocrural joints rotated ∼4° more in the plantar flexion than dorsiflexion phase (p = 0.004; indicated with a cross in Table 2).

## 4 Discussion

Our findings highlight the complex and variable interactions between the bones of the ankle joint complex (talus and calcaneus) during locomotion. Specifically, we aimed to determine the relative contribution of the talocrural joint (talus relative to tibia) to overall ankle joint complex (calcaneus relative to tibia) motion *in vivo.* As previously suggested (e.g., Arndt et al., 2004; de Asla et al., 2006; Kleipool & Blankevoort, 2010; Sheehan, 2010), we have confirmed that the talocrural joint is responsible for the vast majority of sagittal plane ankle joint complex motion (∼80 %). The remaining ∼20 % of ankle joint complex motion in the sagittal plane occurs at the subtalar joint. The substantial amount of dorsi/plantar flexion motion in the subtalar joint is surprising for a joint that was previously believed to be primarily responsible for frontal and transverse plane motion [2], confirming previously conducted BVR studies [13,14,[16], [17], [18],^41^]. In some cases (e.g., subject #9 during hopping), there was equal motion in the sagittal plane at the subtalar joint as in the frontal and transverse planes.

There was no systematic kinematic difference in walking and running gait phases across participants in talocrural and ankle joint complex motion, despite the previously shown differences in energetic and kinematic patterns [[27], [28], [29]]. During hopping, the total rotation of the talocrural and ankle complex was approximately 4° greater in plantar flexion than in dorsiflexion. A slightly larger plantar flexion magnitude together with a longer plantar flexion phase has also been observed in previous publications [42,43], but the mechanism behind this finding remains unknown. We speculate that during dorsiflexion, the soft tissues absorb energy, whereas during plantar flexion, sufficient energy needs to be generated for push-off, hence the larger range of motion in the hindfoot during the plantar flexion phase likely relates to work requirements to maintain constant hop height [44].

In some participants (e.g., Fig. 2, subject #7 during dorsiflexion phase in walking and running), talocrural motion exceeded total ankle joint complex motion (contributions >100 %), suggesting that there was opposite motion occurring at the subtalar joint (e.g., Fig. 2, subject #7 plantar flexion of the subtalar joint). The difference in magnitude between the talocrural joint and total ankle joint complex for total rotation and dorsi flexion motion was about 2°. Regarding out-of-sagittal planes, the difference between talocrural and ankle joint complex motion increased up to ∼6° (e.g., Fig. 3, dorsiflexion phase of running subject eight). Moreover, the talocrural joint displayed substantial motion in the frontal and transverse planes, which, for some participants (e.g., see Fig. 3, subject #1 during plantar flexion phase in walking) [9,10], approached 50 % of the magnitudes observed in the plantar flexion phase of the sagittal plane during walking. We also observed opposing motion in the frontal and transverse planes at the talocrural joint to the overall ankle complex motion, for some individuals (e.g. see Fig. 3, subject #2 during running). These findings indicate that while the entire ankle joint complex moves in one direction, individual joints within the ankle joint complex might not follow the same pattern. This pattern would remain undetected when approximating the entire ankle joint complex as one rigid body in computational models or when using skin-based motion capture systems and traditional bone-pair (Euler-angle based) analysis. Determining these opposing motion patterns may be important to measure underlying soft tissue strain, and potentially joint contact stress at the subtalar joint level which might be a risk indicator for degenerative joint diseases. Our findings are aligned with previous magnetic resonance imaging results showing helical talocrural joint axes of motion during toe-rises are oriented in such manner that rotation occurs in all three cardinal planes [40]. However, we question the interpretation [40], that the talocrural joint can and should be modelled as a fixed hinge joint [29]. Rather, we consider the talocrural joint as a multi-plane joint which is dominant in one plane during sagittal plane-oriented motions such as walking, running, and hopping, but may have sufficient degrees of freedom to allow greater out of sagittal plane motion when required.

The variability of joint contributions to motion across all three planes within the ankle joint complex emphasizes the importance of a personalised (subject-specific) approach to modelling the ankle joint complex. Inter-subject variability of talocrural and ankle joint complex motion across all three planes were large in both their magnitude and direction. Our data shows that the variability was most noticeable in both joints in the frontal and transverse planes. Our findings support previous intra-cortical bone pin studies [[7], [8], [9]] and recent BVR studies [14,17,19,29], adding to the growing body of literature to highlight the large variability in ankle kinematics between participants during locomotion. Variations of motion in these planes may be due to differences in individual bone shapes or soft tissue characteristics driving, guiding, or limiting bony interactions. Another factor might be that the tibia coordinate system is shape-based and individual tibia morphology might emphasize some non-sagittal differences. Inter-individual variability in both joints was also larger in walking and running than for hopping, which might allude to the higher demands to maintain stability compared to hopping on the same spot, or it could stem from the different pools of participants between walking/running and hopping.

From a clinical perspective, personalised approaches may deliver greater resolution to detect pathological motion patterns, as the overall ankle joint complex range of motion might remain unaffected in various conditions, but individual joint contributions might change due to acute or chronic ligamentous or osseous changes. The contribution approach might be included in monitoring treatment and rehabilitation outcomes or in pre- and post-surgery planning stages in addition to previously established kinematics metrics. Additionally, surgeons and clinicians may be better equipped to select the most appropriate treatment options for each patient if they have the resolution to understand function at an individual joint level, using personalised approaches. Furthermore, non-invasive methods (e.g., inertial measurement devices and marker-less motion capture) combined with artificial intelligence (e.g., advanced musculoskeletal modelling) could be alternatives to the approaches presented here. However, we believe the best way forward is to comprehensively understand the role of the talus to the overall ankle joint complex before developing simplified predictive models.

Although highly accurate and non-invasive, BVR is a time-consuming technology. This limits the number of participants and trials from which data can be processed. Further, the capture volume is small, and this presents challenges in data collection. Osseous occlusion can make bone tracking difficult, and a limited field of view can miss some parts of the foot and tibia during touchdown to toe-off during walking and running. Yet, on average, 88 % of the ground contact period during walking and running was tracked (Supplement Fig. S1). In contrast, hopping offers the advantage of a consistent view of the entire foot. The accuracy of the manual tracking procedure for the BVR images was performed by one examiner who was repeatedly trained on a participant with tantalum implanted beads in the foot bones as a gold standard using an openly available dataset (34) until a consistent tracking error margin of 2° for rotations and 1 mm for translations was achieved. We acknowledge that intra-rater reliability for the BVR may be greater than suggested by the training environment, but previous work with the same system showed intra-rater reliability within 1.86 mm for translations and 1.9° for rotations specifically for foot bones (slightly higher for inter-rater reliability at 2.30 mm and 2.6°) [15]. Therefore, we are confident in our statements regarding rotation magnitudes and the conclusions we drew from them. Lastly, combining data from two study protocols could be considered a limitation. However, the similar findings across both data sets strengthen our interpretations of talocrural and subtalar joint contributions due to generalisability across two different participant groups. We consider this a strength of the current manuscript as it confirms findings across different locomotion tasks and participant samples.

## 5 Conclusion

Our study has highlighted that the human ankle joint complex is indeed highly complex and variable in function. While the talocrural joint appears to be the primary contributor to sagittal plane motion, there is also substantial frontal and transverse plane motion occurring within this joint. Considerable between-subject and between-joint variability was observed for frontal and transverse plane motion of the subtalar and talocrural joints. The large variation between individuals may suggest that unique participant morphology could be a crucial factor in the function of the ankle joint complex, along with other factors such as soft tissue characteristics and kinetics. In summary, future research should explore the relationship between morphology, soft tissue function (such as muscle forces, tendon, and ligament properties), and kinematic and kinetic behaviours in the ankle joint complex to enhance the diagnosis and treatment of foot disorders.

## CRediT authorship contribution statement

**Anja-Verena Behling:** Writing – review & editing, Writing – original draft, Visualization, Software, Methodology, Investigation, Funding acquisition, Formal analysis, Conceptualization. **Lauren Welte:** Writing – review & editing, Supervision, Software, Resources, Data curation. **Michael J. Rainbow:** Writing – review & editing, Visualization, Validation, Supervision, Methodology, Data curation, Conceptualization. **Luke Kelly:** Writing – review & editing, Supervision, Methodology, Funding acquisition, Conceptualization.

## Data availability

The data supporting the findings of this study can be requested from the corresponding author.

## Declaration of competing interest

AVB, LW, MR, and LK declare that they have no conflict of interest. AVB is supported by the ISB Matching Dissertation Grant. LW is funded by the Natural Sciences and Engineering Research Council Postdoctoral Fellowship (NSERC PDF: 558140-2021). MR is funded by the 10.13039/501100000038Natural Sciences and Engineering Research Council of Canada Discover Grant (RGPIN/04880-2022) and LK by the Australian Research Council Discovery Early Career Research Award (DE200100585).
