# Supplementary material for: Human in vivo talocrural contributions to ankle joint complex kinematics during walking, running, and hopping
Source: Heliyon. 2024 Dec 19;11(1):e41301. doi: 10.1016/j.heliyon.2024.e41301 (PMC11755030; doi:10.1016/j.heliyon.2024.e41301)
Supplement: Multimedia component 1 [file mmc1.docx]

Supplement Material for

*“*Human *in vivo* talocrural contributions to ankle joint complex kinematics during walking, running, and hopping”

Behling, Anja-Verena^1,2^; Welte, Lauren^3,4^; Rainbow, Michael J Rainbow^2^; Kelly, Luke^1,5^

^1^School of Human Movement and Nutrition Science, The University of Queensland, Brisbane, Australia

^2^Department of Mechanical and Materials Engineering, Queen's University, Kingston, Canada

^3^Department of Mechanical Engineering, University of Alberta, Edmonton, Canada

^4^Department of Biomedical Engineering, University of Alberta, Edmonton, Canada

^5^Griffith Centre of Biomedical & Rehabilitation Engineering, Griffith University, Gold Coast, Australia

[a.behling@uq.edu.au](mailto:a.behling@uq.edu.au)


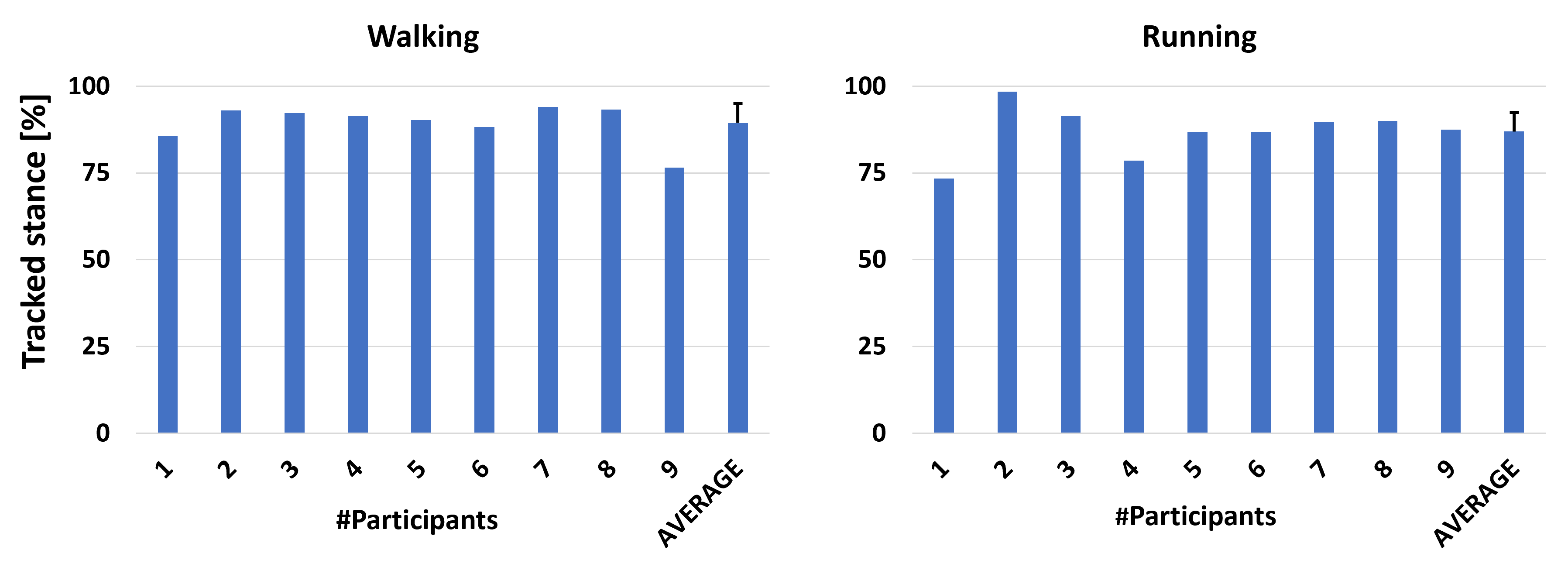


Figure S1: Percent tracked portion of the support phase that was used to calculate the dorsi- and plantar flexion phase of the ankle joint complex (n_Participants_ = 9). Please note, the foot typically left the field of view of the biplanar videoradiography system before the 15N threshold during push-off in vertical GRF.


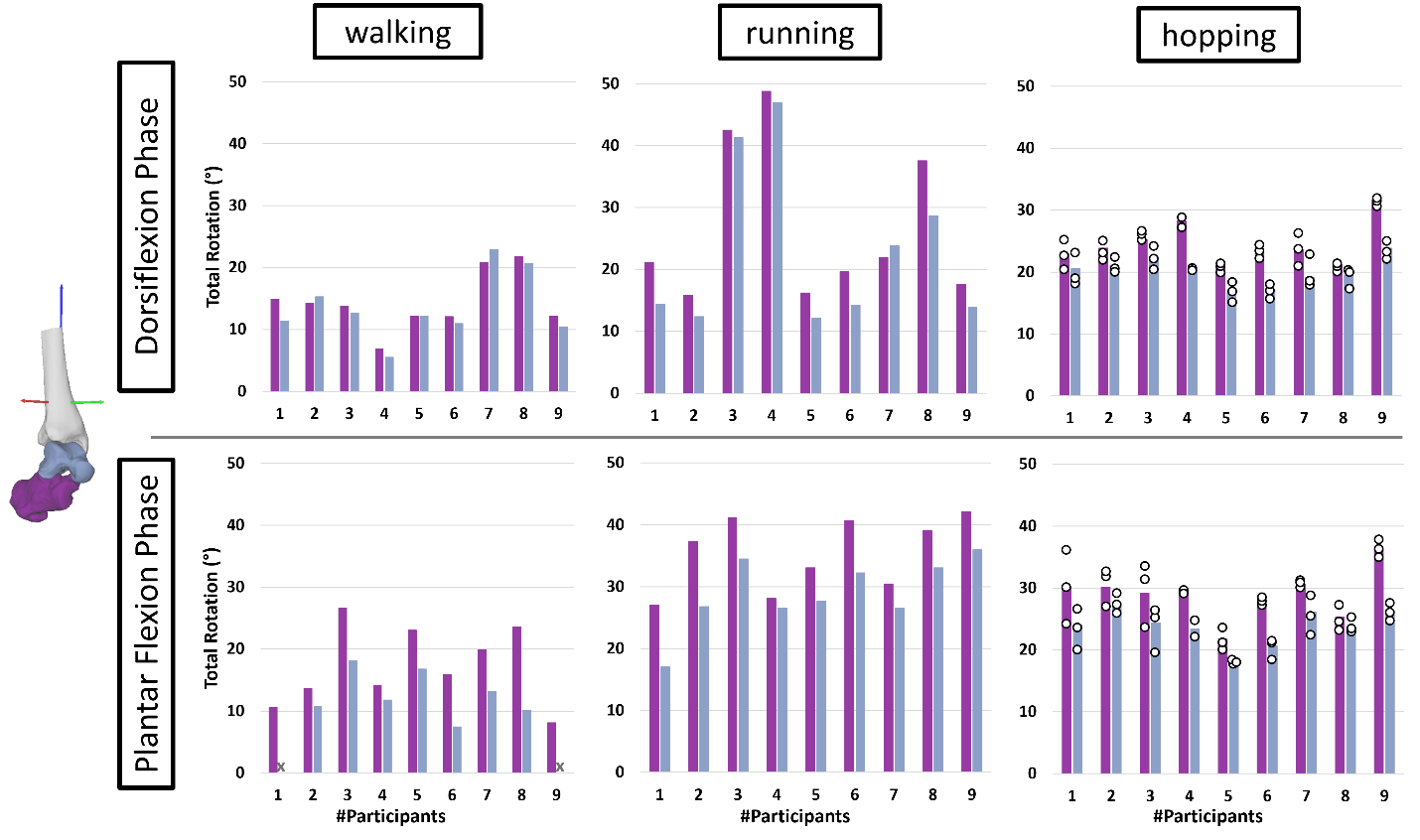


Figure S2: Total rotation magnitudes in degrees during different modes of locomotion for the dorsiflexion (top row) and plantar flexion (bottom row with grey outline) phase for the ankle joint complex (purple) and talocrural joint (blue grey). Subtalar joint motion can be inferred by the difference between the ankle joint complex and the talocrural joint. n_Participants_ = 9; n_Trials_walking/running_ = 1; n_Trials_hopping_ = 3. X indicates a missing value due to total rotation values of <5°. The white circles indicate individual trials for hopping.


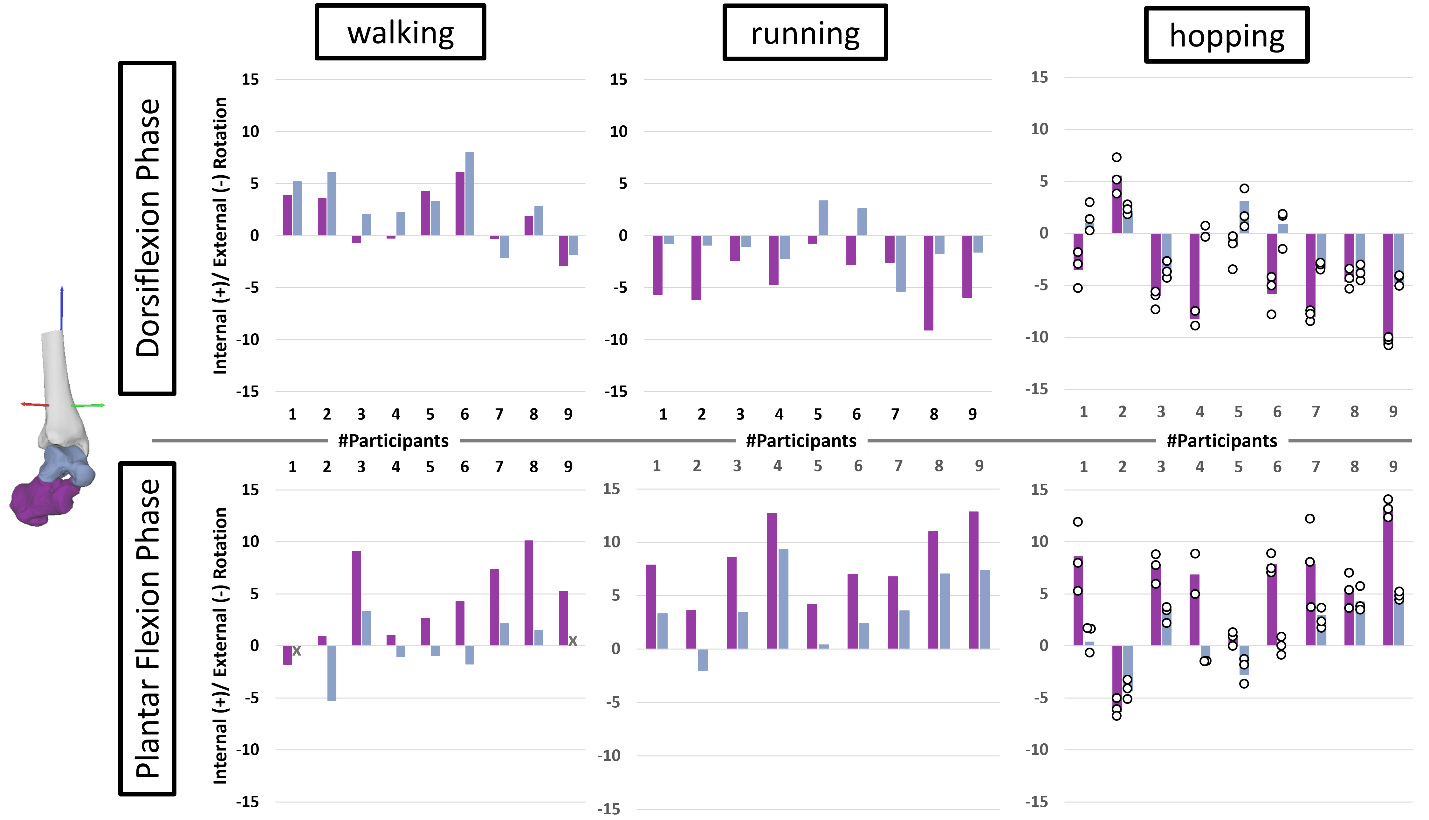


Figure S3: Transverse plane kinematics for talocrural joint (blue grey) and ankle joint complex (purple) joint for all participants (n_Participants_ = 9; n_Trials_walking/running_ = 1; n_Trials_hopping_ = 3) in degrees. Positive and negative rotations are determined based on the right-hand rule (i.e., adduction). The dorsiflexion phase is at the top row while the plantarflexion phase (bottom row) is outlined in grey. X indicates a missing value due to total rotation values of <5°. Subtalar joint motion can be inferred by the difference between the ankle joint complex and the talocrural joint. The white circles indicate individual trials for hopping.
